# Global Deletome Profile of *Saccharomyces cerevisiae* Exposed to the Technology-Critical Element Yttrium

**DOI:** 10.3389/fmicb.2018.02005

**Published:** 2018-09-04

**Authors:** Nicolas Grosjean, Elisabeth M. Gross, Marie Le Jean, Damien Blaudez

**Affiliations:** ^1^Université de Lorraine, CNRS, LIEC, Nancy, France; ^2^Université de Lorraine, CNRS, LIEC, Metz, France

**Keywords:** genome-wide screening, *Saccharomyces cerevisiae*, technology critical element, yeast mutants, yttrium toxicity

## Abstract

The emergence of the technology-critical-element yttrium as a contaminant in the environment raises concern regarding its toxicological impact on living organisms. The molecular mechanisms underlying yttrium toxicity must be delineated. We considered the genomic phenotyping of a mutant collection of *Saccharomyces cerevisiae* to be of particular interest to decipher key cellular pathways involved either in yttrium toxicity or detoxification mechanisms. Among the 4733 mutants exposed to yttrium, 333 exhibited modified growth, of which 56 were sensitive and 277 were resistant. Several functions involved in yttrium toxicity mitigation emerged, primarily vacuolar acidification and retrograde transport. Conversely, functional categories overrepresented in the yttrium toxicity response included cytoskeleton organization and endocytosis, protein transport and vesicle trafficking, lipid metabolism, as well as signaling pathways. Comparison with similar studies carried out using other metals and stressors showed a response pattern similar to nickel stress. One third of the identified mutants highlighted peculiar cellular effects triggered by yttrium, specifically those affecting the pheromone-dependent signaling pathway or sphingolipid metabolic processes. Taken together, these data emphasize the role of the plasma membrane as a hotspot for yttrium toxicity. The up-to-now lack of data concerning yttrium toxicity at the cellular and molecular levels makes this pioneer study using the model *S. cerevisiae* an excellent first basis for the assessment of yttrium toxicity toward eukaryotes.

## Introduction

Mainly mined in China, which possesses the largest deposits, and lower proportions in India, Brazil, and Malaysia, yttrium is a metallic element belonging to the group of rare earth elements (REE). Yttrium is naturally present in the earth crust with an abundance similar to cobalt, zinc, copper, chromium, or lead (Haxel et al., 2002). This element displays unique and essential properties for diverse technologies and is therefore considered a technology-critical element (TCE). The main use of yttrium is for phosphor production (54%) and, to a lesser extent, in white LED lights and ceramic production (32%) (Ciacci et al., 2015). Additional minor uses are also reported, such as additives to improve glass resistance or mechanical properties of alloys, in some lasers, as a catalyst in ethylene polymerization, or in cancer treatment using the radioactive isotope yttrium-90 (Riaz et al., 2014). According to the expansion of these high technology-related needs, annual yttrium production is strikingly rising. It rose from 2400 t/year in 1998 (United States Geological Survey, 2000) to 8000–10,000 t/year in 2015 (United States Geological Survey, 2016). As a consequence, high residual yttrium concentrations (e.g., from 148 to 330 ppm in the A layer and from 60 to 242 ppm in the B layer in soil samples from Chinese mining sites) can be found at REE-mining sites (Wei et al., 2001). Additionally, other anthropogenic activities also contribute to the spreading of yttrium in the environment. Deposits from the combustion of coal still significantly contribute to the dissemination of yttrium worldwide, with concentrations ranging from 94 to 3540 ppm (mean 408) in coal ash and 191–259 ppm in coal fly ash (Mayfield and Lewis, 2013). Moreover, up to 110 ppm yttrium can be found in phosphate rocks (Bunus, 2000). In addition to the increasing demand and spread, another major drawback is the lack of sustainable processes for the recycling of end-use products containing this element. Taken together, these observations suggest that yttrium is an emerging contaminant for the environment (Ciacci et al., 2015).

Few studies are currently available regarding the effect of TCE on the environment or on organisms, despite its increasing abundance. To date, the scarce data concerning yttrium toxicity (Hirano et al., 1993; Li et al., 2015; Zhang et al., 2016; Trifuoggi et al., 2017; Wang Y. M. et al., 2017) mostly focus either on yttrium nanoparticles (Zhang et al., 2016; Zhou et al., 2016) or indirect effects caused by the use of the radioactive isotope yttrium-90 (Riaz et al., 2014). Although yttrium toxicity has been observed for some organisms, the associated detrimental mechanisms remain unknown (Hirano et al., 1993; Li et al., 2015; Zhang et al., 2016; Trifuoggi et al., 2017; Wang Y. M. et al., 2017). Thus, there is a strong interest in unraveling the potential toxicological effects of these elements on well-known organisms to build a general and robust knowledge.

The budding yeast *Saccharomyces cerevisiae* has been widely used as a model to uncover metal toxicity mechanisms as well as pathways that allow eukaryotes to cope with toxic metals. As an example, unprogrammed gene silencing, a major mechanism of nickel toxicity and carcinogenicity in humans, was also identified in *S. cerevisiae* (Lee et al., 1995; Broday et al., 1999; Sutherland et al., 2001; Costa et al., 2005). Those findings further support the use of *S. cerevisiae* as a model organism to elucidate metal toxicity mechanisms in humans due to the high conservation of such mechanisms between these organisms. Genomic phenotyping of knock-out (KO) mutant collections is a method of choice to decipher general functions as well as key genes involved in metal homeostasis in *S. cerevisiae*. This experimental approach has been widely applied, since the KO mutant collection release for various metals such as cadmium (Ruotolo et al., 2008; Serero et al., 2008; Thorsen et al., 2009; Wang, L. et al., 2017), nickel (Ruotolo et al., 2008; Arita et al., 2009; Bleackley et al., 2011), zinc (Pagani et al., 2007; Wang et al., 2007; Jin et al., 2008; Bleackley et al., 2011), aluminum (Kakimoto et al., 2005; Tun et al., 2014), arsenic (Haugen et al., 2004; Thorsen et al., 2006, 2009; Jin et al., 2008; Ruotolo et al., 2008; Serero et al., 2008; Johnson et al., 2016a), copper (Wang et al., 2007; Jo et al., 2008; Bleackley et al., 2011), iron (Wang et al., 2007; Jo et al., 2008; Bleackley et al., 2011), manganese (Wang et al., 2007; Bleackley et al., 2011; Chesi et al., 2012), cobalt (Bleackley et al., 2011), or chromium (Jin et al., 2008; Johnson et al., 2016b) among others. The identified mechanisms thanks to this model organism and approach have provided insights into conserved pathways in other eukaryotes (Botstein et al., 1997; Kachroo et al., 2017).

In this study, the knock-out mutant collection of the haploid strain BY4741 from EUROSCARF was screened to identify both sensitive and resistant mutants to yttrium. The yttrium-specific response was analyzed by comparisons between our set of data with those from previous phenotypic screens on other metals or stressors. The cellular compartments and functions involved in the response to yttrium exposure were investigated in detail, shedding light for the first time on molecular responses toward this emerging contaminant.

## Materials and Methods

### Yeast Strains and Chemicals

The wild-type BY4741 strain of *S. cerevisiae* (MATa his3Δ1 leu2Δ0 met15Δ0 ura3Δ0) and the complete set of 4733 deletion mutants for non-essential genes used in this study were purchased from EUROSCARF (Institute of Molecular Biosciences, Frankfurt, Germany). Mutants of the *S. cerevisiae* BY4742 strain (MATα his3Δ1 leu2Δ0 lys2Δ0 ura3Δ0) were also purchased from EUROSCARF. Yttrium(III) chloride hexahydrate (99.9% purity, #211648) was from Sigma-Aldrich (St. Louis, MO, United States).

### Yttrium Toxicity Screening

The wild-type strain and the deletion mutants, arrayed in 96-well master plates, were grown in 200 μL of YPD (10 g.L^-1^ peptone, 20 g.L^-1^ glucose, and 10 g.L^-1^ yeast extract) medium until stationary phase at 28°C. A first screening for the identification of Y-resistant and Y-sensitive mutants was carried out by comparing their growth (respectively, increased or impaired) to that of the wild-type on a toxic yttrium concentration. In more detail, the strains were pin replicated using a Thermo Scientific^TM^ Nunc^TM^ Replication System (250520) on YPD agar plates or on the same medium supplemented with 3.75 mM yttrium chloride, allowing us to visually identify in a single step both resistant and sensitive strains. Four pin replications were performed for each mutant. Growth was recorded after 5 days at 28°C. In a second screening, the previously selected mutants were individually retested to confirm their sensitive or resistant phenotype by 10-fold serial dilution spot assays. The mutant and the wild-type strains were grown as described above, and 5 μL of stationary cultures were further diluted from 10^0^ to 10^-5^ and spotted on YPD agar plates supplemented with 100% (3.75 mM) and 105% (3.90 mM) of the yttrium concentration used in the first screening. These concentrations were chosen to discriminate the different sensitivity and resistance levels, respectively, as defined below. Growth was recorded after 7 days at 28°C. Sensitivity and resistance levels were assigned to the mutant strains, according to the number of growing dilutions. Consequently, mutant strains exhibiting a reduction in colony-forming ability at the first, second-third, or fourth-fifth dilution were classified as “high” (HS), “medium” (MS), or “low” (LS) sensitive to yttrium, respectively. Conversely, mutant strains exhibiting an increase in colony-forming ability at the second, third-fourth, or fifth-sixth dilution were classified as “low” (LR), “medium” (MR), or “high” (HR) resistant to yttrium, respectively. Finally, a set of 20 mutants, randomly chosen in the different sensitivity/resistance categories, was confirmed in the parent MATα BY4742 background.

### Gene and Function Annotation

Gene descriptions corresponding to the Y-sensitive and Y-resistant mutants were retrieved from the Saccharomyces Genome Database (SGD)^1^. Biological process as well as cellular compartment analyses were performed using KEGG^2^, MIPS, Gene Ontology [GO Term Finder Program (Boyle et al., 2004)] and FunSpec^3^ and evaluated for statistical significance (cutoff: *P* < 0.01). The Y mutant set comparisons with other screenings were carried out by cluster analyses using Cluster 3.0 (Hoon et al., 2004) and Java Treeview 1.1.6r^4^. Hierarchical clustering was performed with the following parameters: average linkage, uncentered correlation, and *k*-mean = 10 when stated. To achieve this analysis, data sets were retrieved from previous genomic phenotyping studies and used if a sufficient number (>50) of mutants was identified. When several independent studies performed similar screenings on the same stressor, the corresponding lists of mutants were combined. Putative Human orthologs of yeast genes and any of their associated OMIM (Hamosh et al., 2005) disease phenotypes were retrieved from the SGD YeastMine platform^5^.

### Functional Enrichment Analysis

The biological pathways that showed enrichment in the yttrium stress condition vs. control were identified using GSEA software v2.0 (Subramanian et al., 2005). The sensitivity and resistance of each mutant was binary encoded to -1 and +1, respectively, for gene ranking. *S. cerevisiae* GO (biological process) derived from the MSigDB format gene-sets list was downloaded from the GO2Msig (Powell, 2014) database and used as a template. The gene set sizes retrieved from GO2Msig datasets were limited to a maximum of 150 and a minimum of 15. Cytoscape v3.4 software (Shannon et al., 2003) and the Enrichment Map v2.1.0 (Merico et al., 2010) plug-in with default settings were used to visualize the GSEA results.

## Results

### Genomic Phenotyping of Yttrium Toxicity

**FIGURE 1:**
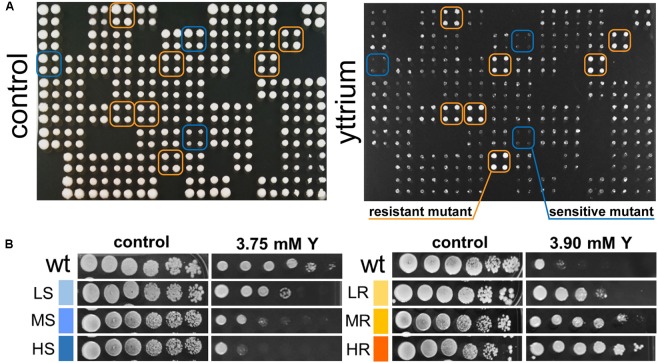
Representative primary screen data for yttrium-responsive mutants and phenotypic confirmation of the identified mutants. **(A)** Representative 384-well format growth test for the yttrium toxicity primary screen (four pin replication of each mutant). A control plate (YPD medium) and the same plate supplemented with 3.75 mM yttrium are shown. Putative yttrium sensitive (blue) and resistant mutants (orange) are highlighted. **(B)** Phenotypic confirmation of selected sensitive and resistant mutants. Wild-type and mutant strains were grown without or with yttrium. Yttrium sensitivity was determined by 10-fold serially diluted spot assays (left to right) with saturation phase grown cells. Mutant strains exhibiting a reduction in colony-forming ability at the first, second-third or fourth-fifth dilution were classified as “high” (HS), “medium” (MS), or “low” (LS) sensitive to yttrium, respectively. Conversely, mutant strains exhibiting an increase in colony-forming ability at the second, third-fourth, or fifth-sixth dilution were classified as “low” (LR), “medium” (MR), or “high” (HR) resistant to yttrium, respectively.

**FIGURE 2:**
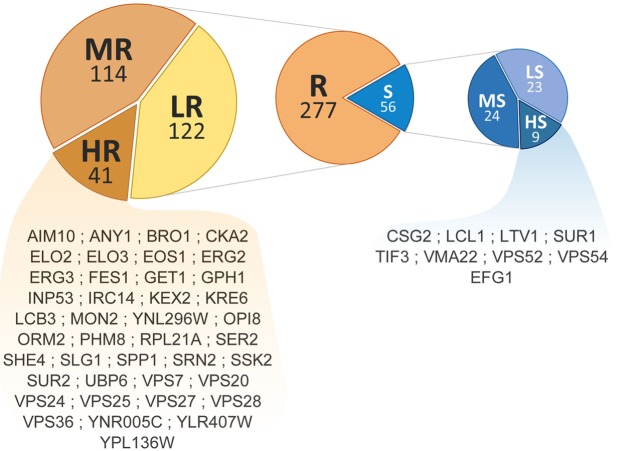
Distribution and ranking of yttrium-responsive mutants. The middle pie chart shows the overall number of resistant (orange) and sensitive (blue) mutants. Detailed proportions of the different categories low (LR), medium (MR), high (HR) resistant mutants (left) and low (LS), medium (MS), and high (HS) sensitive mutants (right) are shown. ORF names of HR and HS mutants are mentioned.

Phenotypic screening of the whole haploid KO mutant collection of *S. cerevisiae* strain BY4741 allowed the identification of mutants with modified growth compared with the wild-type strain when exposed to the TCE yttrium (**Figure 1**). The second screening showed that the number of false positives picked from the first screening was relatively low (10% of the total set) (data not shown). Among the 333 confirmed Y-responsive mutants, 56 were sensitive while five times as many (277) exhibited a resistant phenotype (**Figure 2**). Following the second screening, mutants were classified into six different categories by their degree of sensitivity/resistance relative to the wild-type strain [spanning from highly sensitive (HS) to highly resistant (HR) mutants] (**Figure 1**). Among the resistant mutants, 14.8%, named in **Figure 2**, were HR. The proportion was similar (16.1%) for HS mutants. The number of low (LR) and medium (MR) resistant mutants accounted for 44.0 and 41.2% of the total resistant set, respectively. Similarly, the number of low (LS) and medium (MS) sensitive mutants represented 41.1 and 42.8% of the sensitive mutants, respectively (**Figure 2**). Yttrium sensitivity or resistance phenotypes were confirmed using randomly chosen mutants, by independent serial dilution assays carried out on mutant strains of the opposite mating type (BY4742) (**Supplementary Table 1**). A total of 29 mutants for dubious proteins (8%) and 22 for uncharacterized ORFs (6%) were included in the 333 mutants (**Supplementary Table 2**).

Further examination of the details of cellular compartments related to these mutants allowed the identification of hotspots for either yttrium toxicity or detoxification. From enquiries of the MIPS subcellular localization database, the following subsequent cellular compartments were predominantly represented: endosome (*P* < 1.00e-14, 22 mutants out of 57), vacuole (*P* = 4.07e-05, 26 out of 224), Golgi apparatus (*P* = 4.24e-04, 16 out of 125), endoplasmic reticulum membrane (*P* = 5.29e-03, 14 out of 131), and Golgi-ER transport vesicles (*P* = 9.10e-03, 8 out of 60) (**Table 1**). For the Y-resistant mutants, endosome (*P* = 4.53e-13, 19 out of 57), vacuole (*P* = 2.30e-03, 19 out of 224), and plasma membrane (*P* = 9.25e-03, 15 out of 184) were clearly mostly impacted, while for sensitive mutants, vacuole (*P* = 2.14e-03, 7 out of 224) and Golgi (*P* = 3.38e-03, 5 out of 125) were emphasized (**Table 1**). Interestingly, compartments such as mitochondria and nucleus were not significantly represented (**Table 1**).

**Table 1:** MIPS subcellular compartments involved in the yttrium stress modulation response.

		All mutants		Resistant mutants		Sensitive mutants	
Subcellular compartment	*f*	*p*-value	*k*	*p*-value	*k*	*p*-value	*k*
Endosome	57	<1.00e-14	22	4.53e-13	19	NS	3
Vacuole	224	4.07e-05	26	2.30e-03	19	2.14e-03	7
Golgi	125	4.24e-04	16	NS	11	3.38e-03	5
ER membrane	131	5.*29*e-03	14	NS	11	NS	3
Golgi-ER transport vesicles	60	9.10e-03	8	NS	6	NS	2
Plasma membrane	184	NS	–	9.25e-03	15	NS	–
Nucleus	–	NS	–	NS	–	NS	–
Mitochondria	–	NS	–	NS	–	NS	–
Peroxisomes	–	NS	–	NS	–	NS	–

*k is the number of mutants found during the present screening, and f is the number of proteins gathered under the category mentioned. NS, not significant.*

As a good eukaryotic model, the OMIM human-related diseases tool from the SGD yeastMine platform allowed the identification of human orthologs and associated disease phenotypes. One or more human orthologs were found for up to 188 of the 333 genes (56.5%) for which deletion rendered the mutant strains resistant or sensitive to yttrium. As high as 59 of these genes (17.7%) are involved in human diseases, such as cancer (**Supplementary Table 2**).

### Comparison of Cellular Toxicity Signatures Between Yttrium and Other Metals

**FIGURE 3:**
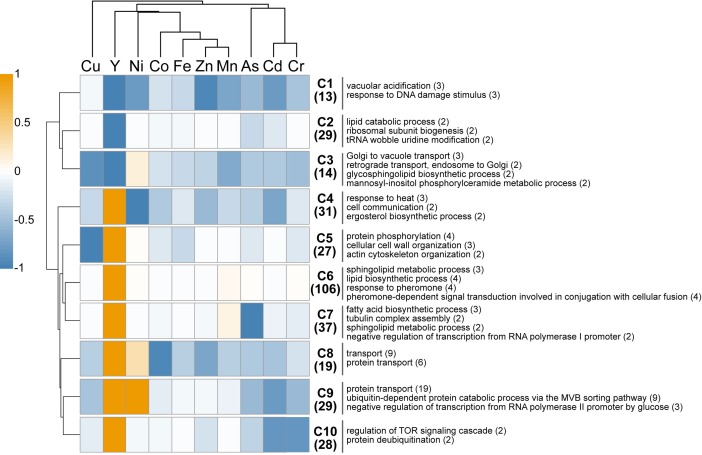
*In silico* comparison of phenotypes of the mutants identified in this study compared with those from other metal-based screening studies. Different colors represent the mean phenotype of the clustered mutants (orange: resistant, blue: sensitive). Functions overrepresented in the clusters (C1–C10) are mentioned on the right. The number of mutants within each cluster or function is specified in brackets. Functions considered were retrieved from FunSpec (biological process GO terms, *P* < 0.01). The hierarchical clustering was done with the following parameters: average linkage, uncentered correlation, *k*-mean = 10.

A comparison of the present Y dataset with other genome-wide deletion mutant screenings carried out on other metals (Cd, Cr, Ni, Cu, Fe, Co, Zn, Mn) and metalloid (As) is provided (**Figure 3**). Excluding Cu, few sensitive mutants (13) grouped together regardless of the metallic species tested, highlighting common response pathways (cluster C1). These shared mutants were notably involved in a global cellular response to metallic stressors such as vacuolar acidification (VMA mutants) and response to DNA damage stimulus. Cluster C3 contained 14 mutants that were sensitive to all metals but Ni, and involved in retrograde transport, Golgi to vacuole transport and glycosphingolipid process. A wide range of resistant mutants (106) were specific to yttrium stress (cluster C6) and belonged to different biological processes spanning the response to pheromone to the sphingolipid metabolic pathway (**Figure 3**).

Regarding mutants with an opposite behavior between yttrium and other metallic species, only three mutants were sensitive to yttrium but resistant toward other metals (ΔSmf2, ΔBud19, and ΔSin4), whereas 179 mutants were resistant to yttrium but sensitive to at least one other metal (clusters C4-C5-C7-C8-C9-C10). Among the latter, several biological processes were represented, mostly belonging to the lipid and fatty acid biosynthetic processes but also to the actin filament/cytoskeleton organization and endocytosis (**Figure 3**). It is noteworthy that the highest similarities were shared between yttrium and nickel (**Figure 3**). Eighty-five mutants displayed a modified phenotype toward both elements, either with the same or opposite behavior. More striking were the 39 mutants that were resistant to both nickel and yttrium (C8-C9) but sensitive to other elements. These mutants belonged mainly to the ubiquitin-dependent protein catabolic process via the multivesicular body (MVB) sorting pathway (*P* < 1.00e-14), including ΔStp22, ΔDoa4, ΔVps25, ΔDid4, ΔSnf7, ΔSrn2, ΔVps36, ΔVps20, ΔSnf8, and ΔVps28. The proteins encoded by these genes are involved in the ESCRT complex and in protein trafficking.

### Comparison of Cellular Toxicity Signatures Between Yttrium and Other Stressors

**FIGURE 4:**
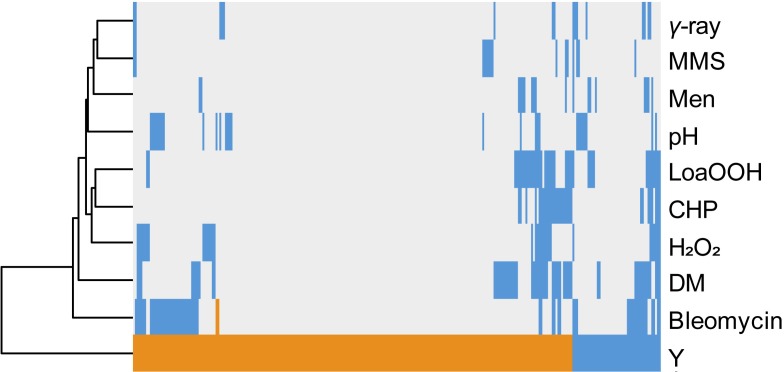
Hierarchical clustering of yttrium sensitivity or resistance-conferring mutations with the mutant sensitivity/resistance profiles of other stressors. The *x*-axis corresponds to gene deletions, and the *y*-axis represents the different physico-chemical stressors. Mutant strains exhibiting either a higher sensitivity, a higher resistance, or no phenotype change when compared to wild-type are shown in blue, orange, and gray, respectively. Non-metal stressors were selected from previous genomic phenotyping screenings conducted on deletion mutant collections. Methyl methane sulfonate (MMS), gamma-radiation (γ-ray), alkaline pH (pH), menadione (Men), hydrogen peroxide (H_2_O_2_), cumene hydroperoxide (CHP), linoleic acid 13-hydroperoxide (LoaOOH), and diamide (DM). Hierarchical clustering was done with the following parameters: average linkage and uncentered correlation.

The same comparative approach was undertaken to investigate putative correlations/convergences between yttrium and gamma-rays, alkaline pH, or oxidative stress induced by a set of chemicals (Bennett et al., 2001; Chang et al., 2002; Aouida et al., 2004; Serrano et al., 2004; Thorpe et al., 2004; **Figure 4**). Interestingly, three different patterns could be observed. First, only a few mutants (40) shared a sensitive phenotype between yttrium and at least one of these other stressors. Second, 115 mutants that were sensitive to those stressors were resistant to yttrium. Third, the majority of the mutants (176) identified in this study were only responding to yttrium (**Figure 4**).

### Mutations Accounting for Yttrium Sensitivity

**FIGURE 5:**
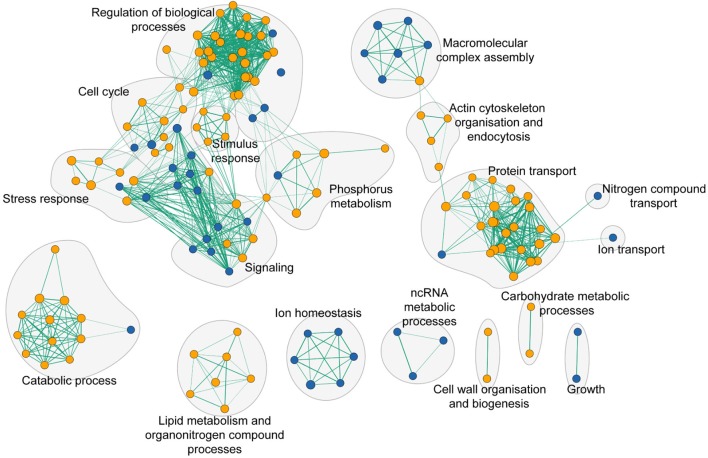
Functional enrichment analysis network of functions that when deleted render cells either sensitive (blue nodes) or resistant (orange nodes) to yttrium. Green lines represent gene overlap between two functions, with the edge width being proportional to the number of shared genes. The enrichment map was built using GSEA and visualized by the enrichment map plugin in Cytoscape.

To assess the global cell response to yttrium, functions involved in yttrium toxicity (resistant mutants) or yttrium resistance (sensitive mutants) mechanisms were revealed by functional enrichment analysis by combining both GO term finder and GSEA approaches (**Figure 5** and **Supplementary Table 3**). In this sub-section, we focus on the description of sensitive mutants, the growth of which was partially or completely inhibited by yttrium exposure. The nine mutants displaying an exacerbated sensitivity (HS mutants) (**Figure 2**) were considered to participate in either the glycosphingolipid metabolic/biosynthetic process (*P* = 3.82e-04) or cellular chemical homeostasis (*P* = 3.82e-03) (**Supplementary Table 4**).

From the entire set of mutants, two mutants (ΔSur1 and ΔCsg2) that were sensitive to yttrium were impaired in the glycosphingolipid biosynthetic process (*P* = 1.96e-04) (**Supplementary Table 3**). Sur1p (also known as Csg1p) is a mannosylinositol phosphorylceramide (MIPC) synthase catalytic subunit that forms a complex with the regulatory subunit Csg2p to act as an inositol phosphorylceramide mannosyltransferase complex.

Ion homeostasis consisted of only sensitive mutants (**Figure 5**). It embodied three different GO terms identified as vacuolar proton-transporting V-type ATPase complex assembly (*P* = 3.90e-04), vacuolar acidification (*P* = 1.18e-03), and ATP hydrolysis coupled proton transport (*P* = 8.25e-03). The only representatives of the VMA family genes were *vma21*, *vma22*, *vma7*, and *vma16* (**Supplementary Table 3**).

**FIGURE 6:**
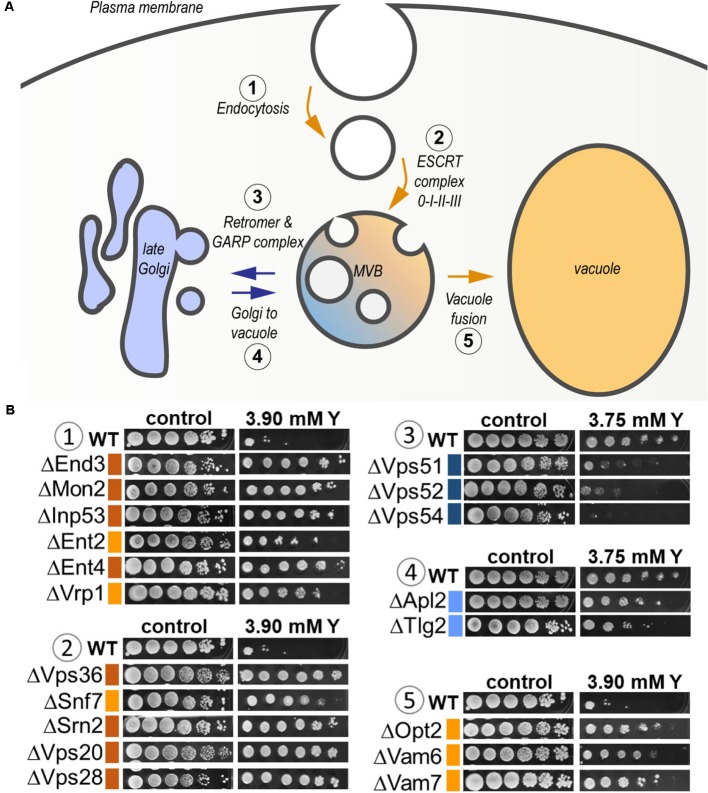
Global representation of yttrium sensitive/resistant mutants involved in the endocytic pathway in *S. cerevisiae*. **(A)** Schematic representation of the endocytic pathway highlighting the yttrium response of KO-mutants involved in the different associated steps. It includes endocytosis (1), targeting to (and formation of) the multivesicular body (MVB) compartment (2), protein retrieval from the MVB to the late Golgi by the Retromer and GARP complex (3), Golgi-to-vacuole trafficking (4), and MVB-to-vacuole fusion (5) pathways. Blue arrows denote the pathways in which the lack of a given protein render cells sensitive to yttrium, while orange arrows correspond to yttrium-resistant mutants. **(B)** Ten-fold dilution drop test assays of mutants for proteins involved in the different mentioned steps (1–5). For the color code of the level of sensitivity or resistance, please refer to the legend of **Figure 1**.

As high as 25% of the total sensitive mutants belonged to the transport function (*P* = 4.79e-03, 14 genes out of 815) (**Supplementary Table 3**). A significant proportion of sensitive mutants consisted of proteins that participate in vesicle transport from both the endosome to the trans-Golgi (*P* = 3.87e-04) and from the Golgi to the vacuole (*P* = 3.76e-05) (**Figure 6** and **Supplementary Table 3**). The GARP (Golgi Associated Retrograde Protein) complex is responsible for vesicles fusion from the endosome to the trans-Golgi (**Figure 6A**). Mutants for *VPS54*, *VPS52*, and *VPS51*, three components of the GARP complex, were particularly sensitive to yttrium (**Figure 6B**). The retrograde transport, which includes the GARP complex, allows the trafficking of extracellular molecules as well as the plasma membrane internalized by endocytosis to the trans-Golgi, thus participating in the recycling of either proteins or lipids from the membrane (**Figure 6A**). Although endocytosis (*P* = 4.36e-03) was also involved, it was represented by only 4 sensitive mutants ΔCdc50, ΔRcy1, ΔVps1, and ΔTlg2 (**Supplementary Table 3**). As part of the SNARE complex (Soluble NSF Attachment Protein Receptor), Tlg2p is also involved in vesicle fusion to the trans-Golgi along with the GARP complex (Burri and Lithgow, 2004; **Figure 6B**). Both Cdc50p and Rcy1p are not only involved in the polarized growth of *S. cerevisiae*, but they also play a role in recycling plasma membrane proteins internalized by endocytosis. In the same way, Vps1p, a dynamin-like GTPase, is required for actin cytoskeleton organization and endocytosis.

Linked to previously unraveled functions, 11 mutants impaired in protein transport (*P* = 1.97e-04) were also sensitive to yttrium. The corresponding proteins were involved at different levels in the protein transport machinery (**Supplementary Table 3**), as illustrated by the following cases. Pep3p as part of the CORVET membrane tethering complex (class C core vacuole/endosome tethering), is involved in vesicular docking fusion together with SNARE protein (Tlg2p), allowing vacuolar biogenesis. The ΔApl2 mutant was also sensitive to yttrium (**Figure 6B**). The latter protein is a subunit of the clathrin-associated protein (AP-1) complex that is involved in clathrin-dependent Golgi protein sorting. Lst4p, for its part, is required for the transport of an amino acid permease from the Golgi to the cell surface. Finally, Cog1p acts in protein trafficking by mediating the fusion of transport vesicles to Golgi compartments.

Among the group of mutants that were identified from the present screening, four mutants for protein components of nuclear and mitochondrial ribosomal subunits displayed a sensitive phenotype (**Supplementary Table 2**). Mrpl27p, a component of the mitochondrial large ribosomal subunit, Rps16ap and Rps19bp, which are part of the nuclear small ribosomal subunit, and Rpp2bp, a component of the ribosomal stalk.

### Mutations Accounting for Yttrium Resistance

Many additional yttrium-resistant over sensitive mutants ensued from the screening. Mutants were considered resistant if their growth was improved compared with the wild-type strain under yttrium exposure. This phenotype indicated a key role of the deleted gene in yttrium cell toxicity since its absence allowed the yeast cells to better cope with this element. Conversely to HS mutants, the 41 HR mutants (**Figure 2**) were related mainly to protein transport with the ubiquitin-dependent protein catabolic process via the MVB sorting pathway (*P* = 1.88e-10) but also to actin cytoskeleton organization (*P* = 1.16e-03), and the sphingolipid biosynthetic process (*P* = 9.60e-05) (**Supplementary Table 4**). On a broader scale, other functions involved in yttrium toxicity were also observed in HR, MR, and LR mutants (**Figure 5** and **Supplementary Table 3**). As observed for sensitive mutants, the transport GO term was highlighted (*P* = 2.55e-04, 54 genes represented out of 815). Hereafter, the major functions retrieved from the analysis of the resistant mutants are detailed.

First, a group constituted predominantly by endocytosis, vesicle trafficking, and actin cytoskeleton organization, could be identified (**Figure 5**). Endocytosis is the process whereby cells recycle membranes or extracellular compounds as well as take up substances or downregulate plasma membrane transporters or receptors. Ten mutants out of 82 (12.1%) for genes involved in endocytosis (*P* = 1.96e-03) were resistant to yttrium (ΔDoa4, ΔYck1, ΔEnt4, ΔEnt2, ΔSla1, ΔVrp1, ΔEnd3, ΔSiw14, ΔMon2, and ΔInp53) (**Supplementary Table 2**).

Second, the functional category “protein transport” was highlighted (**Figure 5** and **Supplementary Table 3**). It included mostly functions involved in localization, transport, and catabolism of proteins (**Supplementary Table 3**). Among the 51 mutants in this category, high representation of the ESCRT complex was apparent (**Figure 6**). Indeed, excluding ΔHse1 and ΔIst1, mutants for all the sub-complexes, known as ESCRT-0 (Vps27p), ESCRT-I (Srn2p, Stp22p, Mvb12p, and Vps28p), ESCRT-II (Vps25p, Snf8p, and Vps36p), ESCRT-III (Did4p, Snf7p, Vps24p, Vps20p, and Did2p), displayed a resistant phenotype (**Figure 6B** and **Supplementary Table 2**). Moreover, the mutants of the ESCRT-associated complex Vta1p-Vps4p and complex Bro1p-Doa4p shared the same phenotype (**Supplementary Table 2**). The ESCRT complex-dependent budding of small vesicles inside the lumen of late endosomes is responsible for the formation of the MVB (**Figure 6A**). The subsequently formed MVB is targeted and fused to the vacuole where the internal vesicles are degraded. This pathway is mostly associated with ubiquitin-dependent protein catabolism. Consistently, two mutants for proteins involved in ubiquitination were resistant to yttrium (**Supplementary Table 2**). On the one hand, Doa4p is a ubiquitin hydrolase that is required for recycling ubiquitin and acts in the late endosome compartment to recover ubiquitin. On the other hand, Ubp2p is a ubiquitin-specific protease that is required for MVB sorting of membrane proteins.

The third group was composed of mutants with disrupted genes encoding enzymes involved in lipid metabolism (**Figure 5**). The sphingolipid metabolic process was the most significant (*P* = 4.46e-05). *DPL1, SUR2, SCS7, LCB3*, and *ARV1* genes are part of the sphingolipid metabolic process, and the corresponding mutants display resistance toward yttrium (**Supplementary Tables 2**, **3**). Closely related functions were also represented, such as sphingolipid biosynthetic process (*TSC3, ELO2, LCB3, ELO3*, and *SUR1*), lipid biosynthetic process (*ELO2, SUR2, CAX4, OAR1, ERG3, ELO3, ERG2, SCS7*, and *ERG24*), and fatty acid biosynthetic process (*ELO2, SUR2, OAR1, ERG3, ELO3*, and *SCS7*) (**Supplementary Table 3**). All these mentioned mutants had impairments in enzymes involved in the modulation of sphingolipid levels, fatty acids synthesis, uptake, and elongation.

A few yttrium-resistant mutants were involved in the transcriptional regulation of RNA polymerase III (*P* = 9.74e-03) (**Supplementary Table 3**). Mutants for *CKB1* and *CKA2*, encoding the catalytic sub-units of casein kinase 2 (CK2), were resistant. Pheromone-dependent signal transduction involved in the conjugation to cellular fusion (*STE50, PTC1, GET3, MFA1, FAR1, STE18, ARV1*, and *STE4*) (*P* = 4.41e-05) and the heterotrimeric G-protein complex cycle (Ste18 and Ste4) (*P* = 7.14e-03) were also found (**Supplementary Table 3**). While these two latter proteins form a dimer that activates the mating signaling pathway, Ste50p acts as an adaptor that links a G protein-associated complex to the effector Ste11p to modulate signal transduction. Get3p amplifies G protein signaling and is also involved in ATP-dependent Golgi to ER trafficking. Mfa1p is the mating pheromone a-factor. Some mutants for proteins involved in the mitogen activated protein kinase signaling cascade (MAPKs) pathway were also resistant to yttrium, including ΔSte50, ΔSte7 (MAPKK), ΔSsk2 (semi-redundant MAPKKK), and a mutant lacking the phosphotyrosine phosphatase Ptp2p that has a role in the inactivation of MAPK. Ptc1p is also a 2C-type protein phosphatase that indirectly inactivates the osmosensing MAPK cascade (Chen and Thorner, 2007).

Finally, mutants for ribosomal sub-units also displayed a resistant phenotype. This category included mutants lacking the mitochondrial large ribosomal subunit components Mrpl23p, Mrpl28p, and Mrpl49p, the large nuclear ribosomal subunit proteins Rpl8bp, Rpl21ap, Rpl37bp, and Rpl40ap, a protein from the small ribosomal subunit Rps12p and Rpp1ap, a component of the ribosomal stalk (**Supplementary Table 2**).

## Discussion

The screening of genome-wide collections of *S. cerevisiae* haploid mutants is a good strategy to uncover essential genes/proteins or functions allowing cells to cope with metal toxicity (Haugen et al., 2004; Kakimoto et al., 2005; Thorsen et al., 2006, 2009; Pagani et al., 2007; Wang et al., 2007; Jin et al., 2008; Jo et al., 2008; Ruotolo et al., 2008; Serero et al., 2008; Arita et al., 2009; Bleackley et al., 2011; Chesi et al., 2012; Tun et al., 2014; Johnson et al., 2016a,b). As a recognized eukaryotic model, *S. cerevisiae* is relevant to better understand the hazardous effects of such contaminants on eukaryotic organisms since many genes are conserved among eukaryotic species. Identified sensitive mutants provide insights into tolerance mechanisms developed by cells, while resistant mutants may shed light on toxicity pathways (e.g., uptake or interacting molecules). In the present study, we adopted this genomic phenotyping approach under exposure to the technological critical element yttrium, an emerging contaminant (United States Geological Survey, 2000; Wei et al., 2001; Mayfield and Lewis, 2013; Ciacci et al., 2015). Strikingly, relatively few mutants (56) showed sensitivity to yttrium. Nonetheless, this number is not abnormal in comparison to other screenings (Kakimoto et al., 2005; Wang et al., 2007; Jin et al., 2008; Tun et al., 2014; Du et al., 2015; Johnson et al., 2016a). The relatively low number of sensitive mutants identified may reflect the restricted number of functions available for yeast cells to cope with yttrium toxicity. In contrast, the high number of resistant mutants is indicative of a wide range of yttrium-induced toxicity mechanisms. Furthermore, comparison of the present mutant set with available studies on other metals outlined the functions and key genes/proteins involved specifically or not in the response of yeast to yttrium stress. These major functions are discussed below.

Mutants for vacuolar membrane ATPase (VMA) family genes were sensitive to yttrium. The VMA allows pH homeostasis by acidification of the vacuolar lumen and thus creates a proton gradient across the tonoplast. As illustrated hereafter, the present set of VMA mutants lacked proteins involved in different steps ranging from assembly, translocation, to the functioning of the VMA. Vma21p and Vma22p are localized to the ER periphery and are involved in VMA assembly before its translocation, first to the Golgi and finally to the vacuolar membrane (Graham et al., 1998). Vma16p encodes the third proteolipid subunit of the VMA (Hirata et al., 1997). It is first located on the ER membrane before being pulled together with the other subunits of the VMA complex (Graham et al., 1998). Vma7p, one of the eight subunits of the V1 peripheral membrane domain of VMA, participates in the structure and activity of the whole complex (Graham et al., 1994; Forgac, 1999). It has been hypothesized that vacuolar membrane vesicles and the VMA-built proton gradient can contribute to the vacuolar uptake of nickel in *S. cerevisiae* (Nishimura et al., 1998). Indeed, the lack of the VMA and its associated proton gradient led to a defect in vacuolar sequestration of nickel and an increased cell sensitivity to this metal (Nishimura et al., 1998). Similar findings have been obtained from most of the other metals tested by genomic phenotyping (Jin et al., 2008; Ruotolo et al., 2008; Bleackley et al., 2011). Moreover, a study based on the functional characterization of VMA has also reported that acidification of the vacuole ensures an important role in ionic (e.g., calcium, manganese, zinc, and copper) homeostasis and detoxification (Ramsay and Gadd, 1997). Thus, VMA activity is likely to act as a general modulation of cellular toxicity induced by metals, including the newly studied element yttrium.

Thirteen mutants for ribosomal proteins, representing 9.5% of the 137 total ribosomal genes of *S. cerevisiae*, were also obtained from the screening, displaying either a sensitive or resistant phenotype toward yttrium exposure. This versatile response of ribosomal proteins has also been previously shown under chromium exposure (Johnson et al., 2016b). The resistant phenotype of the mutated cells could be explained by a strategy of slowing down cell growth through decreased protein synthesis. Hence, cells would mainly shift their metabolism toward toxicity scavenging and cellular components repair. This hypothesis has recently been reinforced by Pan et al. (2017), who recorded a down-regulation of ribosomal gene expression under cadmium stress, concluding that the cellular resources could be diverted to the metal stress response rather than being consumed for the synthesis of ribosomes. Conversely, the explanation for the sensitive phenotype displayed by some ribosome-related mutants is not as straightforward. It has been further argued that chromium might impact protein translation (Johnson et al., 2016b). Altogether, this finding and the present data indicate that some of these ribosomal proteins would be essential for cells to cope with chromium and yttrium stress.

In addition to the impact of yttrium toxicity at the translational level, this study highlighted the putative role of several signaling pathways for modulation of the yttrium response. First, two mutants, ΔCkb1 and ΔCka2, showed a resistance phenotype to yttrium. CK2 is a Ser/Thr protein kinase that has many substrates, including transcription factors and all RNA polymerases. Our results suggest that this enzyme might regulate yttrium toxicity signaling. The ΔCkb1 mutant was also resistant to Cr(III) and was associated with a reduced accumulation compared with wild-type [16% less Cr(III) after 16 h], while the ΔCka2 mutant accumulated more Cr(III) (Johnson et al., 2016b). The involvement of CK2 in the response to aluminum, chromium, and arsenic toxicity underlines its key role in metal toxicity mitigation via the signaling pathway, possibly by regulating cell entry of toxic metals (Johnson et al., 2016a,b), including yttrium. Another aspect of signaling arose, since several genes involved in the pheromone-dependent signal transduction as well as in the MAPK signaling cascade conferred, when mutated, an yttrium-resistant phenotype to cells. Activation of the MAPK signaling cascade in response to different signals or stresses, such as exposure to metals, is well known in *S. cerevisiae* (Chen and Thorner, 2007). Surprisingly, discrepancies were observed for these pathways when comparing yttrium exposure to zinc, arsenic, and cadmium stresses. Indeed, these mutants, except ΔBck1, were resistant to yttrium but sensitive to the other metals (Jin et al., 2008). Among the MAPK signal transduction pathways, the pheromone response pathway was represented along with the Ste4p and Ste18p components of the heterotrimeric G-protein complex, which is associated with pheromone receptors (Chen and Thorner, 2007). It is well established that pheromone stimulation leads to cell cycle arrest. Moreover, it has been suggested that this pathway facilitates cell wall alterations required for the mating process (Chen and Thorner, 2007) and thus leads to a more susceptible cell wall. Therefore, the resistance phenotype of mutants disrupted in the pheromone-dependent signal transduction pathway could be explained by the lack of cell cycle arrest or by the reduction of cell wall alterations, allowing cells to cope with yttrium toxicity.

Comparison of the dataset of yttrium-sensitive and -resistant mutants with those from other genomic phenotyping screenings on gamma-rays, alkaline pH, or oxidative stress induced by a set of chemicals (Bennett et al., 2001; Chang et al., 2002; Aouida et al., 2004; Serrano et al., 2004; Thorpe et al., 2004), displayed limited similarities. These results do not support the hypothesis of the involvement of oxidative stress, DNA-damage or of an alkaline-pH-related response in yttrium toxicity. This hypothesis is reinforced by the observation that neither mitochondrial nor nuclear compartments were significantly impaired by yttrium (**Table 1**).

The disruption of genes related to protein transport and vesicular trafficking modified the cell response to yttrium. Notably, the whole cascade starting from membrane endocytosis to the trans-Golgi and vacuole, through endosomes and the MVB, was represented among the mutants impaired by yttrium. Interestingly, protein transport as well as recycling or catabolism via the ubiquitin-dependent catabolic process was central to the yeast response to yttrium. This phenomenon has only been previously evidenced for nickel and methyl-mercury, since the deletion of MVB sorting pathway-related proteins (ESCRT complex) rendered yeast highly resistant to these stressors (Ruotolo et al., 2008; Arita et al., 2009; Hwang et al., 2014). Our results suggest a similar conclusion for yttrium since mutants from the same pathway were also resistant to yttrium. Conversely, mutants for three components of the retrograde pathway, namely, Vps54p, Vps52p, and Vps51p, were all sensitive to yttrium, cadmium (Ruotolo et al., 2008), arsenic (Thorsen et al., 2009; Johnson et al., 2016b), and chromium (Johnson et al., 2016b), but not to nickel (Ruotolo et al., 2008). However, no straightforward explanation could be drawn. Nevertheless, the contrasting phenotypes for mutants of the ESCRT-complex vs. the retrograde pathway particularly suggest that the MVB could be a central target for yttrium toxicity modulation.

The actin cytoskeleton along with endocytosis appears to be also of high importance in yttrium homeostasis. Indeed, sub-functions related to these two categories are highly represented among yttrium-resistant mutants. The cytoskeleton and endocytosis are closely related as the latter is governed by actin (Robertson et al., 2009). Interestingly, mutants for the majority of genes involved in the connection between the cytoskeleton and endocytosis were yttrium-resistant, such as ΔVrp1, ΔEnd3, ΔSla1, ΔEnt2, and ΔEnt4. Both Vrp1p (verprolin) and End3p promote actin nucleation and cytoskeleton organization and endocytosis (Benedetti et al., 1994; Munn et al., 1995). Sla1p plays a crucial role in the cortical actin patch structure and organization. It interacts with End3p and links the actin cytoskeleton and the endocytosis machinery (Warren et al., 2002). Finally, both Ent2p and Ent4p, mammalian epsin-like proteins, possess an Epsin N-terminal homology domain that is usually found in proteins implicated in endocytosis regulation or cytoskeleton organization. It is noteworthy that endocytosis has only been previously mentioned as taking part in chromate homeostasis (Holland and Avery, 2009). Chromate accumulation and cell sensitivity were higher in endocytosis-defective mutants than in wild-type. Moreover, it has been demonstrated that chromate stress increases endocytosis in *S. cerevisiae*. Taken together, these data emphasize that actin-related endocytosis functions as a compulsory mechanism for chromate resistance, possibly through endocytic inactivation of Cr-transporting proteins. However, endocytosis mutants were yttrium-resistant, suggesting the presence of different homeostasis mechanisms between yttrium and chromate.

The toxicity of a metal might begin with the prerequisite step of its entry into cells. Thus, decreased or suppressed uptake of yttrium should lead to cellular resistance toward this element. Two hypotheses can be drawn to explain this resistance phenotype: (i) The improper recycling of a yet unidentified efflux system for yttrium would drive its accumulation at the plasma membrane. Consequently, yttrium detoxification in the cytosol would be enhanced through increased efflux of this element out of the cell. Endocytosis of metal transporters has been shown in different organisms to modulate the uptake of specific metals, rationalizing this hypothesis. For instance, the yeast membrane metal transporters Zrt1p and Ctr1p are post-translationally regulated by ubiquitination and endocytosis to be degraded in the vacuole (Ooi et al., 1996; Gitan and Eide, 2000; Liu et al., 2007). In agreement with this hypothesis, defects in these two proteins (Doa4p and Ubp2p) involved in ubiquitination induced resistance to yttrium. (ii) Alternatively, endocytic vesicles containing yttrium (either membrane-bound or in the vesicle lumen) could be involved in the non-specific uptake of yttrium. According to these two hypotheses, mutations in either of these two transport systems would result in a high intracellular content of yttrium. However, putative non-specific plasma membrane transporters mediating yttrium uptake cannot be ruled out, as suggested for nickel (Ruotolo et al., 2008). To test these hypotheses, further proteomic and ionomic (yttrium quantification) studies are needed.

The lipid biosynthetic process was identified in the present screening by fatty acid, ergosterol, and sphingolipid synthesis mutants that displayed yttrium resistance. Hence, the biosynthesis of these molecules appears to trigger yttrium toxicity, excluding in two mutants, ΔCsg2 and ΔSur1, which were highly sensitive to yttrium as well as to calcium (Beeler et al., 1994, 1997). In the latter case, a calcium-induced plasma membrane alteration would lead to sensitivity of these mutants. The complex formed by the MIPC synthase catalytic sub-unit Sur1p and the regulatory sub-unit Csg2p acts as an inositol phosphorylceramide mannosyltransferase complex (Beeler et al., 1997). Additionally, ΔCsg2 and ΔSur1 also accumulate fewer mannosylated sphingolipids along with an increase in lysophosphatidylinositol compared with the wild-type strain (Beeler et al., 1997). The calcium sensitivity of these mutants would result from the accumulation and/or mislocalization of IPC (inositol-P-ceramide) rather than a depletion of MIPC content. MIPC has been shown to be required for the proper localization of proteins to the plasma-membrane in *Schizosaccharomyces pombe* (Nakase et al., 2010). Interestingly, some other studies have proposed that this lipid might be involved in determining the activity of the plasma membrane drug transporters and/or the permeability properties of this membrane (Lisman et al., 2004). Therefore, an imbalance in plasma membrane sphingolipid composition could be responsible for the yttrium-sensitive phenotype displayed by these mutants. Moreover, several mutants mentioned earlier lack enzymes that participate either in fatty acid biosynthesis, uptake, or elongation and subsequently impact cellular sphingolipid levels. Sphingolipids are implicated in numerous pathways from temperature tolerance to the cell cycle, actin metabolism, and protein stability and localization (Montefusco et al., 2014). It has been demonstrated that several classes of lipids participate in endocytosis, and more particularly, in the clathrin-independent endocytic pathway (Kirkham and Parton, 2005). As demonstrated in Chinese hamster ovary cells, different endocytic pathways are altered by the depletion of either general sphingolipids or, more specifically, glycosphingolipids (Cheng et al., 2006). Taken together, these data emphasize the role of the plasma membrane as a hotspot for yttrium toxicity.

## Conclusion

The present study reports the first cellular and molecular insights addressing yttrium toxicity and thus shedding light on the previously unknown impact of yttrium on eukaryotes. Both cellular toxicity and detoxification mechanisms revealed in *S. cerevisiae* provide valuable information for further potential environmental issues derived from this emerging metallic contaminant. The cellular response to yttrium appeared to exhibit peculiarities in comparison to other metals. Signaling pathways, protein translation, and the vacuolar compartment are well known for their involvement in metal toxicity modulation, while unexpected pathways such as endocytosis and sphingolipid metabolism revealed new perspectives in our understanding of metal homeostasis in eukaryotes. Based on the present findings, further investigations must be conducted in other eukaryotes, including human cells. Such studies have been performed using nickel and demonstrated a high degree of conservation of various aspects of metal toxicity between *S. cerevisiae* and mammals (Lee et al., 1995; Broday et al., 1999; Sutherland et al., 2001; Costa et al., 2005). This information will aid in deciphering whether the identified mechanisms of yttrium toxicity can be extended to all eukaryotes.

## Author Contributions

NG, MLJ, and DB designed the research and analyzed the data. NG performed the research. NG, MLJ, EG, and DB evaluated the data and wrote the manuscript. All authors read and approved the final manuscript.

## Conflict of Interest Statement

The authors declare that the research was conducted in the absence of any commercial or financial relationships that could be construed as a potential conflict of interest.

## Footnotes

1 http://www.yeastgenome.org

2 http://www.genome.jp/kegg/

3 http://funspec.med.utoronto.ca

4 http://jtreeview.sourceforge.net

5 http://yeastmine.yeastgenome.org/yeastmine/
